# Role of Exact Exchange and Empirical Dispersion in Density Functional Theory-Based Three-Body Noncovalent Interactions

**DOI:** 10.1021/acs.jpca.4c03262

**Published:** 2024-09-25

**Authors:** Mauricio Cafiero

**Affiliations:** Department of Chemistry, University of Reading, Whiteknights Campus, Reading RG6 6UR, U.K.

## Abstract

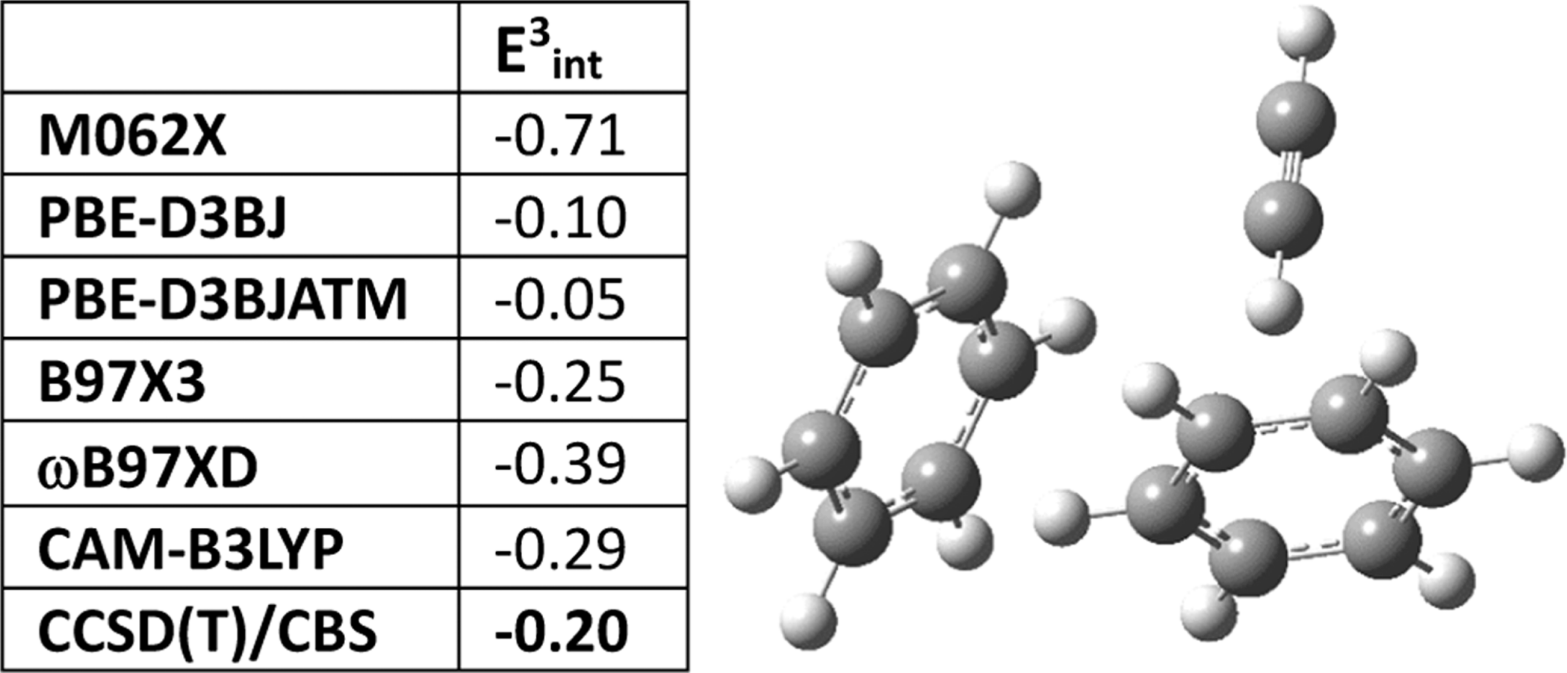

Total and three-body interaction energies are calculated for a benchmark set of three-body systems using a range of different types of density functional theory (DFT) methods, with the results compared to CCSD(T)/CBS results from the benchmark reference [*Phys. Chem. Chem. Phys.***2023**, *25*, 28621–28637]. Inclusion of Hartree-Fock exchange, via either a global or range-separated hybrid approach or inclusion of empirical dispersion corrections, increases accuracy for total and three-body interactions. Basis set convergence testing shows that the aug-cc-pVTZ basis set is well converged with little to no change seen when using quadruple-ζ basis sets. The accuracy of the DFT methods is similar when calculating interaction energies for both global and local minimum structures. Overall, the CAM-B3LYP-D3BJ, B97D3, and ωB97XD functionals are recommended for calculating three-body interactions.

## 1 Introduction

**Figure 1:**
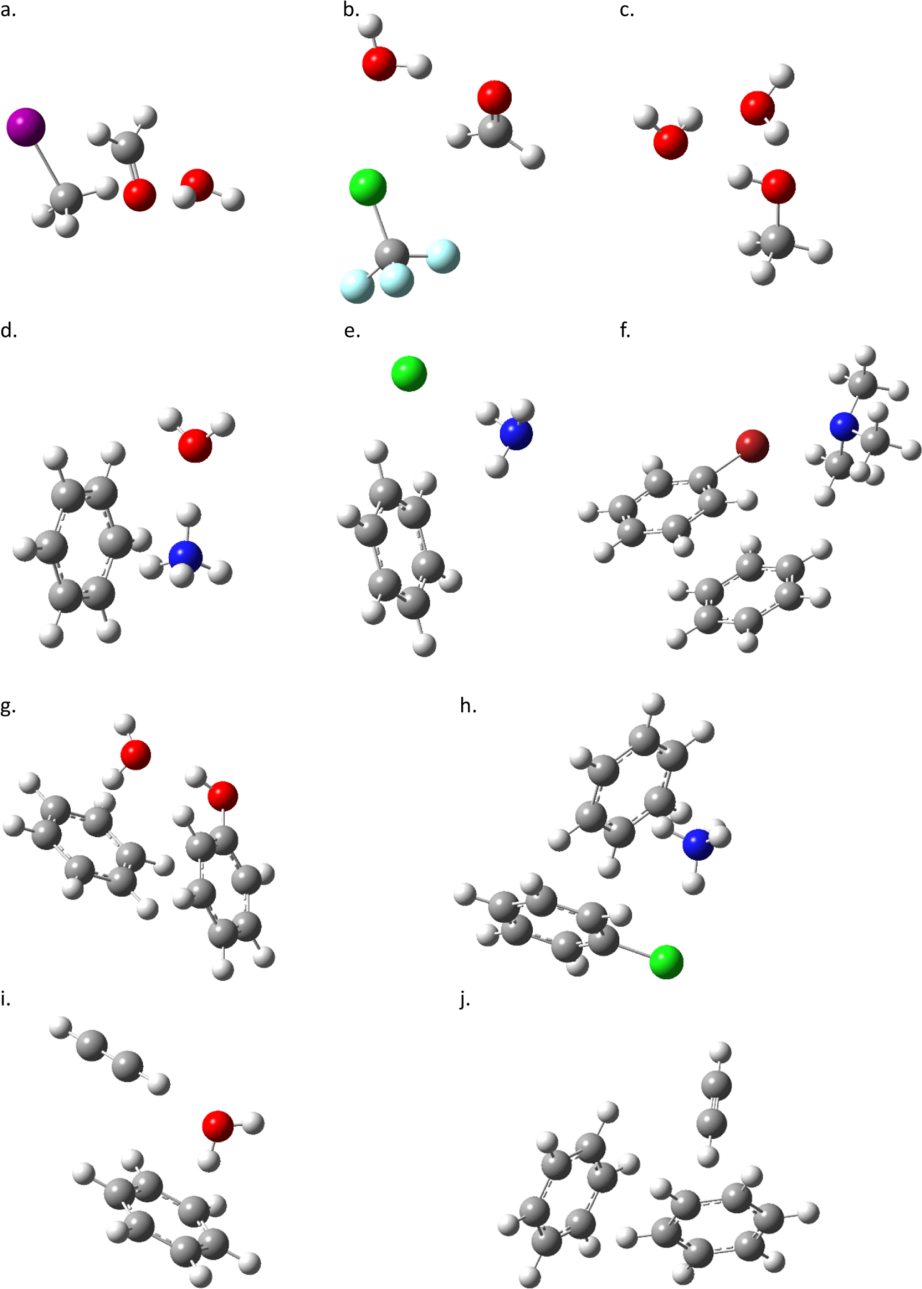
Optimized global minimum structures:^2^ (a) CH_3_I–H_2_CO–H_2_O, (b) CF_3_Cl–H_2_CO–H_2_O, (c) CH_3_OH–H_2_O–H_2_O, (d) NH_4_^+^–C_6_H_6_–H_2_O, (e) Cl^–^–NH_3_–C_6_H_6_, (f) C_6_H_5_Br–(CH_3_)_3_N–C_6_H_6_, (g) C_6_H_5_OH–C_6_H_6_–H_2_O, (h) C_6_H_5_Cl–C_6_H_6_–NH_4_^+^, (i) HCCH–C_6_H_6_–H_2_O, and (j) HCCH–C_6_H_6_–C_6_H_6_.

**Figure 2:**
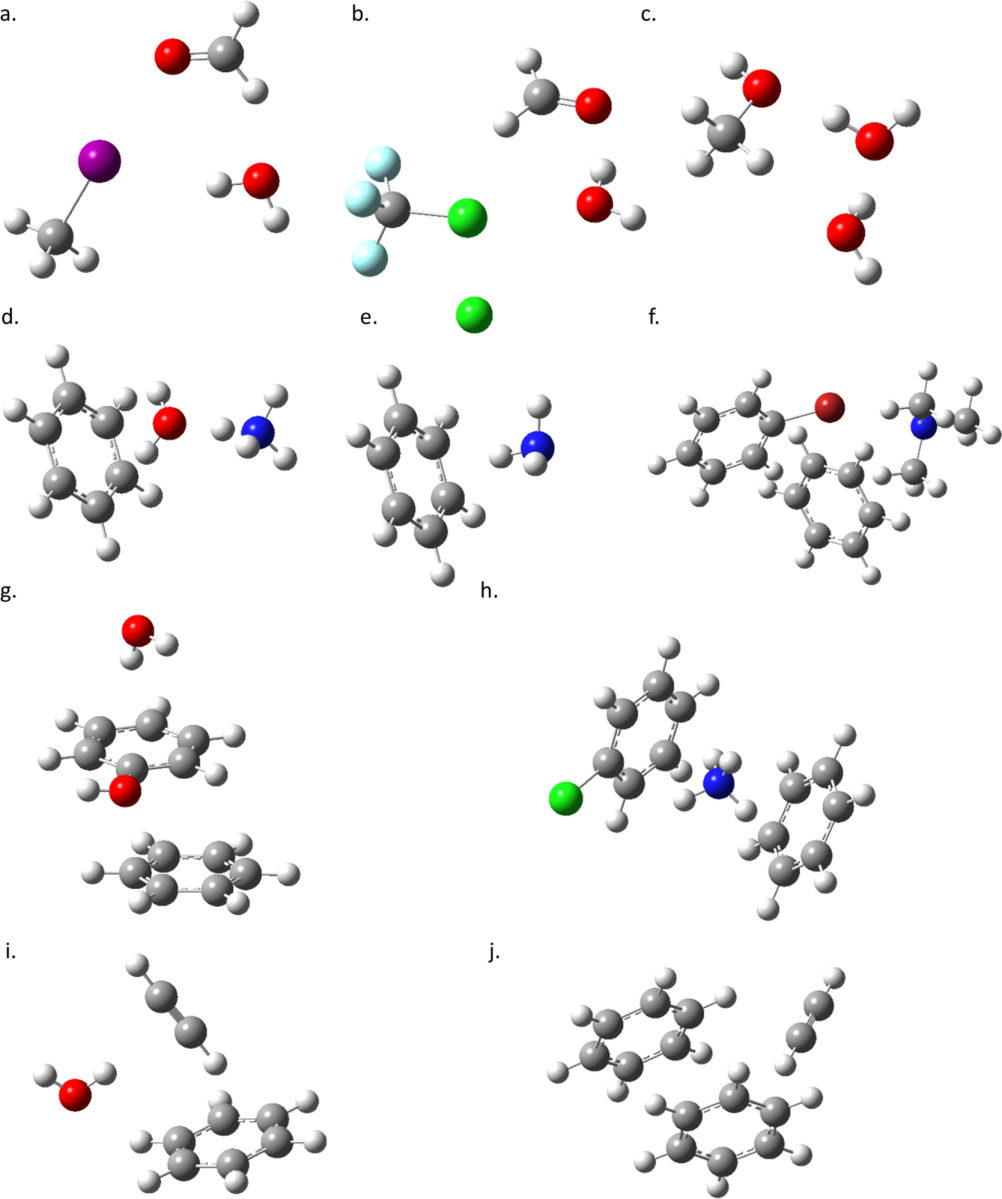
Optimized local minimum structures:^2^ (a) CH_3_I–H_2_CO–H_2_O, (b) CF_3_Cl–H_2_CO–H_2_O, (c) CH_3_OH–H_2_O–H_2_O, (d) NH_4_^+^–C_6_H_6_–H_2_O, (e) Cl^–^–NH_3_–C_6_H_6_, (f) C_6_H_5_Br–(CH_3_)_3_N–C_6_H_6_, (g) C_6_H_5_OH–C_6_H_6_–H_2_O, (h) C_6_H_5_Cl–C_6_H_6_–NH_4_^+^, (i) HCCH–C_6_H_6_–H_2_O, and (j) HCCH–C_6_H_6_–C_6_H_6_.

In recent work, the current author studied the importance of three-body interaction energy terms when deconstructing density functional theory (DFT)-based protein–ligand binding energies as a sum on *n*-body terms.^1^ It was found that the magnitude of the sum of three-body interaction energies can total 2–30% of the sum of the two-body interaction energies and can account for 2–30% of the total interaction energy, depending on the functional used.^1^ Thus, DFT methods that can accurately compute three-body interactions are needed for protein–ligand binding studies, such as those used in drug design. Ochieng and Patkowski recently published an excellent database of 20 three-body complexes with benchmark structures and CCSD(T)/CBS interaction energies.^2^ The complexes in that work (see Figures 1 and 2) are comprised of polar molecules including carbonyl, amine, and hydroxyl groups, ions, benzene rings, and substituted phenyl rings. Furthermore, the rings are found in both sandwich and T-shaped conformations. The pairwise and three-body interactions between all of these molecules form a fair representation of the interactions found between amino acid residues in a protein, as well as typical interactions between those residues and bound ligands. Examples of these interactions in a protein–ligand systems as well as a discussion of the three body effects can be found in the current author’s recent work.^1^ In this work, the benchmarks of Ochieng and co-workers will be used to evaluate a range of DFT methods for their accuracy in three-body interactions. Few other benchmark databases for three-body systems have been published. The 3B-69 database of Řezáč and co-workers is comprised of 69 trimers, all with three identical monomers,^3^ which is not readily applicable to protein–ligand binding. Likewise, the S22(3) database of Alkan and co-workers contains trimers with up to two unique monomers (AAA and AAB type trimers).^4^ The database of Low et al. does contain trimer complexes relevant to protein–ligand binding, but uses an MP2-based method for computing reference energies.^5^ The advantage that the Ochieng and Patkowski benchmark database has over these is that it has at least two and up to three distinct monomers in the complex, has well-established, high accuracy benchmark interaction energy values, and covers a wide variety of intermolecular forces that are important in bioactive complexes.

Noncovalent interactions calculated with DFT typically rely on empirical dispersion terms added to an existing functional, the most widely used form of which is due to Grimme.^6−8^ The D2 version of this correction includes a “C6” dispersion term which takes into account only pairwise, dipole/dipole interactions. The D3 flavor of this correction includes a “C8” term to account forpairwise dipole/quadrupole interactions, as well as a proper three-body “C9” term in the D3^ATM^ extension of this model. Anatole Von Lilienfeld and Tkatchenko studied the contributions of novel *atomic* three-body dispersion energy terms, including C6 and C9 terms, to noncovalent interactions of several biologically relevant systems in the S22 database, as well as other systems such as drug-DNA binding, base-pair interactions, and aromatic clusters.^9^ Their results were proven to be more accurate than the original D3 corrections. In that work, they found that the three-body contributions can range from 14% to 51% of the total interaction, though it is typically lower than 15%. Petersson et al. built on the D3 model to include *anisotropic* two and three-body dispersion corrections (including C6 and C9 terms).^10^ This work shows that the anisotropic terms can reduce error relative to that of CCSD(T)/CBS by 75%. A study by Jankiewicz et al. argues that DFT with dispersion corrections is less accurate than DFT without dispersion interactions.^11^ The current author’s recent work^1^ shows that, in some cases, a D2 or D3 correction does not improve a functional’s performance for noncovalent interactions, such as with the Minnesota functionals which incorporate dispersion well without corrections,^12,13^ but in other cases it does offer improvement.

Schröder et al. studied the interplay of the coefficients used in the dispersion functional form (such as the *C*_6_^*AB*^ and terms) and the damping functional form (such as S_8_, a_1_, and a_2_).^14^ Specifically, they examined the popular Becke–Johnson damping scheme and found that the C8 term can be excluded entirely via a reparameterization of the functional and damping scheme that reduced the coefficients from three to one. The following year, Smith et al. showed a more conventional reparameterization of the damping coefficients with additional training data that reduced errors greatly for some functionals.^15^

Hapka et al. performed a decomposition of the noncovalent energy of clusters and found that the most important component of the three-body energy is the *nonadditive exchange*, which they showed can be a large fraction of the total interaction energy and larger by far than the three-body dispersion.^16^ In that work, they showed that range-separated DFT methods, which incorporate 100% exact exchange in specific regions, had the best performance. This is in agreement with the author’s previous work^1^ as well as the current work, which will show that two of the three most accurate DFT methods for three-body interactions are range-separated.

As shown in the work cited above and in recent work by the current author, the two indicators of the accuracy that a DFT method will have for three-body interactions are the amount of *nonlocality* in the functional, as expressed by the amount of *exact (HF) exchange*, and empirical dispersion corrections.^1^ Thus, the DFT methods chosen for study in this work are “families” of methods with increasing amounts of nonlocality. For example, the progression from M06L to M06 to M06-2X adds 27% and 54% HF exchange in the first two steps,^12,13^ and then the M06-2X-D3^7,8^ method adds empirical dispersion to that.

The decomposition of interaction energies into components has been outlined by Xantheas^17^ and Ucisik et al.,^18^ as well as by the current author.^1^ A two-body interaction energy, Δ^2^*E*(*i*,*j*), can be defined as
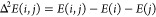
1where *E*(*i*,*j*) is the energy of the complex of the *i*-th and *j*-th components, *E*(*i*) is the energy of the *i*-th component, and *E*(*j*) is the energy of the *j*-th component. Similarly, a three-body interaction energy, Δ^3^*E*(*i*,*j*,l), can be defined as
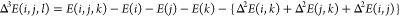
2where *E*(*i*,*j*,*k*) is the energy of the three-body complex of components *i*, *j*, and *k*, and Δ^2^*E*(*i*,*j*) is the interaction energy of the complex of components *i* and *j*. The terms in the braces subtract the two body energies from the total so that only the truly three-body effects remain. Relating this to the formalism of Ochieng and Patkowski,^2^ we have
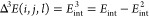
3And
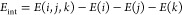
4In this work, *E*_int_ and *E*_int_^3^ will be reported, and *E*_int_^2^ can be calculated from these two quantities.

## 2 Computational Methods

The structures of 20 molecular complexes were taken from the benchmark work of Ochieng and Patkowski.^2^ These 20 complexes are made up of ten unique molecular complexes of biochemical relevance, which each have a global minimum structure (Figure 1) and a local minimum structure (Figure 2) with prominent intermolecular forces different from the global minimum. The ten global minimum structures were used for the broad evaluation of the ability of DFT methods and basis sets to accurately model the three-body energies, and the ten local minimum structures were used with only the four most accurate DFT methods and one basis set.

The ten global minimum structures were evaluated against the benchmark CCSD(T)/CBS three-body energies with 16 DFT methods. The 16 DFT methods were comprised of five “families” of methods with different amounts of nonlocality in the form of exact exchange and empirical dispersion: BLYP^19,20^ → B3LYP^21^ → CAM-B3LYP^22^ → CAM-B3LYP-D3BJ,^6^ M06L^13^ → M06^12^ → M06-2X^12^ → M06-2X-D3,^6^ PBE^23^ → HSE^24^ → PBE-D3BJ,^7^ HCTH^25^ → τHCTH^26^ → τHCTHhyb,^26^ and B97D3^7^ → ωB97XD.^27^ It should be noted that the CAM-B3LYP and PBE functionals with D3 dispersion use the Becke–Johnson damping formulation, while the M062X functional uses the original D3 damping. All D3 corrections used include only pairwise C6 and C8 terms, with the exception of a subset of complexes studied with PBE-D3BJ*ATM*,^29^ which includes three-body terms in the empirical dispersion calculation. Each of the 16 DFT-based three-body energies were calculated with the aug-cc-pVTZ basis set,^29,30^ and the CAM-B3LYP-D3BJ, M06-2X, B97D3, ωB97XD, and PBE-D3BJ three-body energies were also calculated with the aug-cc-pVQZ^29,30^ and def2-QZVPP^31^ basis sets to test for basis set convergence. In the case of DFT, where energies do not necessarily converge with the completeness of the basis set, this basis-set testing ensures that the basis set in question is large and flexible enough to adequately describe the electron density (i.e., a 3-21G basis set would yield dramatically different results). By testing the augmented, triple-ζ results against two different flavors of augmented quadruple-ζ basis sets, the completeness of the electron density description can be demonstrated. Furthermore, if one is not using the same basis that was used for the training of the DFT functional, this type of testing ensures the stability of the chosen basis set. The ten local minimum structures were evaluated against the benchmark CCSD(T)/CBS three-body energies with five DFT methods (CAM-B3LYP-D3BJ, M06-2X, B97D3, ωB97XD, and PBE-D3BJ) and the def2-QZVPP basis set.

The DFT functionals used in this work take into account three-body interactions in several ways. The most direct accounting of three body interactions is through *nonadditive exchange energy*. As detailed in the work by Hapka et al.,^16^ nonadditive exchange includes three-body and higher interactions and is not explicitly accounted for in pure DFT functionals. Exact (HF) exchange, though, does account for nonadditive exchange, and so hybrid DFT functionals are better than pure functionals at replicating this term. This means that hybrid functionals should provide better total three-body interaction energies and better three-body-only interaction energies (that is, with all two-body contributions subtracted). This has been demonstrated quantitatively by Hapka et al.^16^ The second accounting for three-body interactions is with empirical dispersion. While the empirical dispersion corrections used in this work include only pairwise contributions (C6 and C8 terms), these can indirectly improve total three-body interactions through interactions such as fragment A polarizes fragment B, which then has a strong interaction with fragment C. These pairwise terms cannot, however, improve three-body-only interactions. A test set of complexes will have three-body empirical dispersion corrections [Axilrod–Teller–Muto (ATM)] calculated below to demonstrate the fact that they do not contribute significantly to the energies studied here. The third way that DFT functionals can take into account the three-body interactions is through training and parametrization, such as in the case of the M06 family of functionals.^12,13,32^

Basis set superposition errors (BSSEs) for the two and three-body calculations were corrected using the counterpoise method,^33^ with orbitals and DFT grid points on the ghost atoms. For the three-body interaction energies, eq 4, all of the atoms on components *j* and *k* were made ghost atoms in the calculation of the energy of the *i-*th component, etc. For the two-body interaction energies needed to calculate Δ^3^*E*(*i*,*j*,*l*) (eq 2), only atoms on component *j* were made into ghost atoms for the calculation of the *i-*th component, that is, the third component in the complex (the *k-*th component) was not included in the counterpoise calculation. Recent work from the current author has shown that for two-body energies, this *local* counterpoise correction accounts for most of the BSSE in DFT/aug-cc-pVDZ calculations, and a *global* counterpoise (including the *k*-th component) is not necessary.^1^ Since this work uses larger basis sets than the referenced work, the quality of the *local* counterpoise correction should be even higher.

The model systems studied in this work (Figures 1 and 2) provide an excellent range of three-body interactions to test the DFT methods. The first three complexes are dominated by dipole interactions, and so the molecular “triple dipole” type of interactions first studied on the atomic level by Axilrod and Teller^28^ can be examined. The next six complexes include polar and charged molecules (and one atomic ion) interacting with π-systems. These are good test systems for ion-dipole–quadrupole, dipole–dipole–quadrupole, and dipole–quadrupole–quadrupole interactions, which may also be called dipole−π and ion−π interactions. The final system is a quadrupole–quadrupole–quadrupole or π–stacking interaction. Between all of these complexes, most interactions found in protein–ligand complexes are represented, as interactions such as ion-dipole and dipole–dipole are subsets of the interactions in the first three complexes. Furthermore, the global and local minimum structures provide different arrangements of these interactions, so while there are 10 systems total, there are 20 different sets of interactions.

All calculations were performed using Gaussian 16,^34^ other than the PBE-D3BJATM calculations, which were performed using the Psi4 program.^35^

## 3 Results and Discussion

### 3.1 Global Minimum Structures

Tables 1–5 show the total interaction energies and the three-body interaction energies (*E*_int_ and *E*_int_^3^) for the ten global minimum structures in the database calculated with each family of DFT methods, compared to the CCSD(T)/CBS reference values. Table 6 shows a summary of the mean absolute errors (MAEs) for all of the DFT methods studied compared with the CCSD(T) standard.

**Table 1:** Total Interaction and Three-Body Interaction Energies for a Benchmark Set of 10 Global Minimum Molecular Complexes Calculated with the M06 Family of Functionals and the aug-cc-pVTZ Basis Set, Compared to the CCSD(T) Reference Values^2^^a^

	reference^2^		M06L		M06		M06-2X		M06-2X-D3	
	*E*_int_	*E*_int_^3^	*E*_int_	*E*_int_^3^	*E*_int_	*E*_int_^3^	*E*_int_	*E*_int_^3^	*E*_int_	*E*_int_^3^
CH_3_I–H_2_CO–H_2_O	–10.31	–0.56	–9.64	–1.28	–9.96	–1.38	–11.06	–0.84	–11.20	–0.84
CF_3_Cl–H_2_CO–H_2_O	–8.70	–0.41	–7.41	–0.94	–7.66	–1.06	–8.79	–0.70	–8.98	–0.70
CH_3_OH–H_2_O–H_2_O	–16.99	–2.42	–16.29	–3.23	–16.44	–3.25	–17.62	–2.72	–17.76	–2.72
NH_4_^+^–C_6_H_6_–H_2_O	–36.23	3.39	–34.85	3.12	–34.99	3.18	–37.10	3.31	–37.46	3.31
Cl^–^–NH_3_–C_6_H_6_	–17.55	1.34	–16.53	0.78	–16.99	0.94	–17.82	1.10	–18.07	1.10
C_6_H_5_Br–(CH_3_)_3_N–C_6_H_6_	–6.86	0.35	–5.04	–0.64	–4.44	–0.49	–6.12	–0.28	–7.28	–0.27
C_6_H_5_OH–C_6_H_6_–H_2_O	–14.53	–1.56	–12.92	–2.24	–12.89	–2.36	–15.48	–2.02	–16.25	–2.02
C_6_H_5_Cl–C_6_H_6_–NH_4_^+^	–33.79	3.79	–31.95	3.14	–32.00	3.30	–34.59	3.46	–35.48	3.46
HCCH–C_6_H_6_–H_2_O	–7.93	–0.67	–6.89	–1.22	–6.87	–1.18	–8.65	–1.01	–8.97	–1.01
HCCH–C_6_H_6_–C_6_H_6_	–6.95	–0.20	–5.03	–1.03	–4.79	–1.02	–7.15	–0.71	–7.91	–0.71

a Values are in kcal/mol.

**Table 2:** Total Interaction and Three-Body Interaction Energies for a Benchmark Set of 10 Global Minimum Molecular Complexes Calculated with the BLYP Family of Functionals and the aug-cc-pVTZ Basis Set, Compared to the CCSD(T) Reference Values^2^^a^

	reference^2^		BLYP		B3LYP		CAM-B3LYP		CAM-B3LYP-D3BJ	
	*E*_int_	*E*_int_^3^	*E*_int_	*E*_int_^3^	*E*_int_	*E*_int_^3^	*E*_int_	*E*_int_^3^	*E*_int_	*E*_int_^3^
CH_3_I–H_2_CO–H_2_O	–10.31	–0.56	–5.05	–0.98	–6.47	–0.79	–8.47	–0.63	–11.12	–0.63
CF_3_Cl–H_2_CO–H_2_O	–8.70	–0.41	–3.65	–0.56	–5.15	–0.49	–7.15	–0.42	–9.04	–0.42
CH_3_OH–H_2_O–H_2_O	–16.99	–2.42	–12.87	–2.78	–14.46	–2.61	–16.93	–2.45	–18.30	–2.45
NH_4_^+^–C_6_H_6_–H_2_O	–36.23	3.39	–29.41	3.65	–31.65	3.62	–34.28	3.63	–37.68	3.63
Cl^–^–NH_3_–C_6_H_6_	–17.55	1.34	–10.95	1.29	–12.48	1.32	–14.29	1.37	–16.92	1.37
C_6_H_5_Br–(CH_3_)_3_N–C_6_H_6_	–6.86	0.35	10.83	0.19	8.30	0.25	4.70	0.29	–5.25	0.29
C_6_H_5_OH–C_6_H_6_–H_2_O	–14.53	–1.56	–1.15	–1.98	–4.03	–1.86	–7.76	–1.74	–14.31	–1.74
C_6_H_5_Cl–C_6_H_6_–NH_4_^+^	–33.79	3.79	–20.26	4.22	–23.10	4.15	–26.68	4.10	–34.56	4.11
HCCH–C_6_H_6_–H_2_O	–7.93	–0.67	–1.00	–0.83	–2.82	–0.77	–4.94	–0.73	–8.09	–0.73
HCCH–C_6_H_6_–C_6_H_6_	–6.95	–0.20	6.13	–0.42	3.58	–0.35	0.51	–0.30	–6.22	–0.29

a Values are in kcal/mol.

**Table 3:** Total Interaction and Three-Body Interaction Energies for a Benchmark Set of 10 Global Minimum Molecular Complexes Calculated with the PBE Family of Functionals and the aug-cc-pVTZ Basis Set, Compared to the CCSD(T) Reference Values^2^^a^

	reference^2^		PBE		HSE		PBE-D3BJ	
	*E*_int_	*E*_int_^3^	*E*_int_	*E*_int_^3^	*E*_int_	*E*_int_^3^	*E*_int_	*E*_int_^3^
CH_3_I–H_2_CO–H_2_O	–10.31	–0.56	–8.52	–0.67	–8.67	–0.62	–11.31	–0.67
CF_3_Cl–H_2_CO–H_2_O	–8.70	–0.41	–6.28	–0.28	–6.65	–0.34	–8.35	–0.28
CH_3_OH–H_2_O–H_2_O	–16.99	–2.42	–16.00	–2.49	–16.41	–2.50	–17.77	–2.49
NH_4_^+^–C_6_H_6_–H_2_O	–36.23	3.39	–34.09	3.96	–35.03	3.79	–37.95	3.96
Cl^–^–NH_3_–C_6_H_6_	–17.55	1.34	–15.11	1.71	–15.33	1.54	–17.90	1.72
C_6_H_5_Br–(CH_3_)_3_N–C_6_H_6_	–6.86	0.35	3.49	0.51	3.00	0.40	–6.13	0.51
C_6_H_5_OH–C_6_H_6_–H_2_O	–14.53	–1.56	–7.66	–1.64	–8.70	–1.70	–14.57	–1.64
C_6_H_5_Cl–C_6_H_6_–NH_4_^+^	–33.79	3.79	–27.62	4.55	–28.56	4.28	–35.93	4.55
HCCH–C_6_H_6_–H_2_O	–7.93	–0.67	–4.90	–0.56	–5.47	–0.64	–8.32	–0.56
HCCH–C_6_H_6_–C_6_H_6_	–6.95	–0.20	0.23	–0.11	–0.71	–0.20	–6.62	–0.11

a Values are in kcal/mol.

**Table 4:** Total Interaction and Three-Body Interaction Energies for a Benchmark Set of 10 Global Minimum Molecular Complexes Calculated with the HCTH Family of Functionals and the aug-cc-pVTZ Basis Set, Compared to the CCSD(T) Reference Values^2^^a^

	reference^2^		HCTH		τHCTH		τHCTHhyb	
	*E*_int_	*E*_int_^3^	*E*_int_	*E*_int_^3^	*E*_int_	*E*_int_^3^	*E*_int_	*E*_int_^3^
CH_3_I–H_2_CO–H_2_O	–10.31	–0.56	–5.80	–0.10	–5.94	–0.48	–7.47	–0.73
CF_3_Cl–H_2_CO–H_2_O	–8.70	–0.41	–4.70	0.22	–4.14	–0.11	–5.50	–0.43
CH_3_OH–H_2_O–H_2_O	–16.99	–2.42	–11.78	–1.93	–13.42	–2.45	–15.05	–2.68
NH_4_^+^–C_6_H_6_–H_2_O	–36.23	3.39	–29.84	4.33	–31.38	4.09	–33.29	3.74
Cl^–^–NH_3_–C_6_H_6_	–17.55	1.34	–12.01	2.14	–12.86	1.87	–14.28	1.48
C_6_H_5_Br–(CH_3_)_3_N–C_6_H_6_	–6.86	0.35	9.98	1.25	9.24	0.80	4.72	0.33
C_6_H_5_OH–C_6_H_6_–H_2_O	–14.53	–1.56	–1.33	–0.93	–2.59	–1.39	–6.64	–1.76
C_6_H_5_Cl–C_6_H_6_–NH_4_^+^	–33.79	3.79	–20.10	5.16	–22.23	4.80	–26.26	4.24
HCCH–C_6_H_6_–H_2_O	–7.93	–0.67	–2.32	–0.08	–2.30	–0.38	–4.17	–0.69
HCCH–C_6_H_6_–C_6_H_6_	–6.95	–0.20	4.52	0.50	4.31	0.13	1.04	–0.25

a Values are in kcal/mol.

**Table 5:** Total Interaction and Three-Body Interaction Energies for a Benchmark Set of 10 Global Minimum Molecular Complexes Calculated with the B97 Family of Functionals and the aug-cc-pVTZ Basis Set, Compared to the CCSD(T) Reference Values^2^^a^

	reference^2^		B97D3		ωB97XD	
	*E*_int_	*E*_int_^3^	*E*_int_	*E*_int_^3^	*E*_int_	*E*_int_^3^
CH_3_I–H_2_CO–H_2_O	–10.31	–0.56	–10.01	–0.81	–9.92	–0.79
CF_3_Cl–H_2_CO–H_2_O	–8.70	–0.41	–6.89	–0.44	–7.39	–0.60
CH_3_OH–H_2_O–H_2_O	–16.99	–2.42	–15.13	–2.83	–16.74	–2.76
NH_4_^+^–C_6_H_6_–H_2_O	–36.23	3.39	–36.69	3.77	–36.85	3.40
Cl^–^–NH_3_–C_6_H_6_	–17.55	1.34	–16.58	1.40	–16.61	1.22
C_6_H_5_Br–(CH_3_)_3_N–C_6_H_6_	–6.86	0.35	–7.23	0.36	–5.88	0.10
C_6_H_5_OH–C_6_H_6_–H_2_O	–14.53	–1.56	–14.10	–1.81	–14.69	–1.85
C_6_H_5_Cl–C_6_H_6_–NH_4_^+^	–33.79	3.79	–36.30	4.34	–34.64	3.83
HCCH–C_6_H_6_–H_2_O	–7.93	–0.67	–7.62	–0.69	–8.14	–0.83
HCCH–C_6_H_6_–C_6_H_6_	–6.95	–0.20	–7.25	–0.252	–7.085	–0.39

a Values are in kcal/mol.

The Minnesota functionals perform well across the board for both the total and the three-body interactions, although it is clear that the accuracy increases with added exact exchange from M06L to M06 to M06-2X. Interestingly, for these functionals, the addition of empirical dispersion decreases the accuracy of M06-2X for the total interaction but does not affect the three-body interaction accuracy at all. While M06-2X has the second lowest MAE of any method studied here for the total interaction energy, its accuracy for the three-body interaction is surpassed by ten of the 12 non-Minnesota methods. Looking at the values in Table 1, it can be seen that the larger errors for the three-body interactions for these functionals come from the complexes with benzene and substituted benzene rings (the fifth through ninth complexes in the table). In the case of C_6_H_5_Br–(CH_3_)_3_N–C_6_H_6_, the three-body interaction energy is *qualitatively* incorrect for all Minnesota functionals, as the functionals are overestimating the attractive force. In fact, in all cases, the DFT three-body interactions are more attractive than the reference CCSD(T) interactions, whereas the total interaction energy is less attractive than the reference energy for the M06L and M06 functionals and only more attractive for the M06-2X and M06-2X-D3 functionals. This would imply that for the two functionals with less exact exchange, longer-range, three-body forces are overestimated compared to the overall forces, while for the two functionals with more exact exchange, all forces are overestimated.

The BLYP-based functionals show a striking decrease in MAE for the total interactions with added nonlocality, with the error going from 9.2 kcal/mol for BLYP to 0.8 kcal/mol for CAM-B3LYP-D3BJ (Table 6). The MAE for the three-body interaction, however, does decrease from BLYP to B3LYP and to CAM-B3LYP, but does not decrease further with the addition of empirical dispersion to CAM-B3LYP. Thus, for both the M06L and BLYP-based functional families, the addition of empirical dispersion makes a difference to total interactions but not to three-body interactions. Table 2 shows that BLYP, B3LYP, and CAM-B3LYP can be qualitatively incorrect for several total interaction energies [C_6_H_5_Br–(CH_3_)_3_N–C_6_H_6_ and HCCH–C_6_H_6_–C_6_H_6_], though they are qualitatively correct for all three-body interactions. This is likely due to the fact that the main overall interactions for these two complexes are attractive π–π and CH–π forces, which cannot be modeled accurately by the BLYP-based functionals without the addition of dispersion corrections, thus leading to incorrect predictions. The three-body interactions for these complexes (dipole–dipole–quadrupole and quadrupole–quadrupole–quadrupole) are dominated by nonadditive exchange as they can be largely described by functionals with exact exchange. In this case, BLYP is an overestimation, and the addition of exchange by B3LYP and CAM-B3LYP brings the three-body interaction closer to the CCSD(T) reference values, but addition of dispersion by CAM-B3LYP-D3BJ does not improve the values further.

The PBE-based functionals have poor accuracy for total interaction energies (Table 6) compared to PBE-D3BJ, which is the third most accurate functional in this category. While the dispersion correction does make the calculation of total interactions more accurate, it does not increase the accuracy of the three-body interaction at all compared to the base-PBE functional. The HSE06 functional, however, does show much better accuracy for three-body interactions than the other two PBE-based functionals, though the accuracy for total interaction energies is poor. As with the BLYP-based methods, PBE is qualitatively incorrect for the total interaction energies for the C_6_H_5_Br–(CH_3_)_3_N–C_6_H_6_ and HCCH–C_6_H_6_–C_6_H_6_ complexes, and while HSE does show the correct qualitative behavior for the HCCH–C_6_H_6_–C_6_H_6_ complex, the results are in error by about 90% compared to the reference (Table 3). While the PBE-D3BJ MAE for the three-body interactions is higher than other functionals, all three-body interactions with this functional are qualitatively correct.

The HCTH-based functionals perform poorly in all cases for total interaction energies (Table 4), though the three-body interaction MAE for τHCTHhyb is in-line with the better-performing DFT methods studied here (Table 6). Comparing the errors for τHCTH and τHCTHhyb shows that it is the HF exchange that leads to greater increased accuracy rather than the kinetic energy density.

**Table 6:** MAE for Total Interaction and Three-Body Interaction Energies for a Benchmark Set of 10 Global Minimum Molecular Complexes Calculated with 16 DFT Functionals and the aug-cc-pVTZ Basis Set, Compared to the CCSD(T) Reference Values^2^^a^

	*E*_int_	*E*_int_^3^
M06L	1.328	0.658
M06	1.279	0.636
M062X	0.6	0.344
M062X-D	0.954	0.344
BLYP	9.246	0.262
B3LYP	7.156	0.178
CAM-B3LYP	4.454	0.108
CAM-B3LYP-D	0.803	0.108
HCTH	8.645	0.752
τHCTH	7.852	0.389
τHCTHhyb	5.295	0.167
B97D3	0.932	0.203
ωB97XD	0.585	0.181
PBE	4.388	0.244
HSE06	3.731	0.152
PBE-D3BJ	0.783	0.245

a Values are in kcal/mol.

The B97-based functionals are the most accurate functionals studied here when both total interaction and three-body interactions are taken into account (Tables 5 and 6). The range-separated ωB97XD has slightly better performance in both categories than B97D3, in line with the improvement in going from B3LYP to CAM-B3LYP, suggesting that introduction of HF exchange via range-separation and global hybrids is a valid approach to increasing total and three-body interaction energy accuracy.

Overall, the M06-2X, M06-2X-D3, CAM-B3LYP-D3BJ, PBE-D3BJ, B97D3, and ωB97XD functionals are the most accurate for total interactions, while B3LYP, CAM-B3LYP-D3BJ, τHCTHhyb, B97D3, ωB97XD, and HSE06 are the most accurate functionals for three-body interactions. In nearly all of these cases, inclusion of HF exchange is needed for accuracy, although the PBE-D3BJ and B97D3 functionals have good accuracy without HF exchange; this is then due to the inclusion of empirical dispersion, which also provides some long-range information in lieu of HF exchange. It should be noted that the dispersion corrections for PBE and CAM-B3LYP do improve the accuracy of the total interaction dramatically but have no effect on the accuracy of the three-body terms. This is due to the D3BJ correction used in both cases including only pairwise C6 and C8 terms and not including three-body C9 or higher-order terms. Taking the intersection of these two sets, CAM-B3LYP-D3BJ, B97D3, and ωB97XD perform well in all cases and are recommended for total and three-body accuracy.

Tables S1 and S7 in the Supporting Information include a column of MAEs for each global (Table S1) and local (Table S7) minimum complex averaged over the five DFT methods used to study the local minima. For global minima, the total interaction MAE is 0.741 kcal/mol, and the three-body-only interaction MAE is 0.216 kcal/mol. For local minima, the MAEs for total and three-body interactions are 0.715 and 0.177 kcal/mol. It can be seen that the DFT methods produce similar accuracy for both the global and the local minimum structures. The largest errors for global and local minimum total interactions were for NH_4_^+^–C_6_H_6_–H_2_O and C_6_H_5_Cl–C_6_H_6_–NH_4_^+^, which both include an unsubstituted benzene, a polar molecule, and an ammonium. The largest errors for the global and local minimum three-body-only interactions were also for the same two complexes, suggesting that the ammonium, paired with the other molecules, may be the common thread that links the complexes with less accurate performance.

The complexes with the smallest total interaction errors for global *and* local minima were HCCH–C_6_H_6_–H_2_O and HCCH–C_6_H_6_–C_6_H_6_. The complexes with the smallest global minimum three-body-only interaction errors were CF_3_Cl–H_2_CO–H_2_O and HCCH–C_6_H_6_–H_2_O, while for the local minimum structures, the smallest three-body only errors were for the complexes CF_3_Cl–H_2_CO–H_2_O and C_6_H_5_OH–C_6_H_6_–H_2_O. There is no common thread among the four complexes with the lowest errors for the global and local minima, which range from dipole–dipole–dipole to quadrupole–quadrupole–quadrupole interactions, and so it may be concluded that while DFT can accurately model a range of three-body systems, systems containing a benzene and a polar molecule *and* an ammonium can be less accurate. This is important to consider, as protonated amines are quite prominent in protein structures.

### 3.2 Basis Set Convergence

Basis set convergence was tested on five widely different functionals selected from the M06, BLYP, B97, and PBE families (CAM-B3LYP-D3BJ, M06-2X, B97D3, ωB97XD, and PBE-D3BJ). These functionals are chosen due to good accuracy for global minima and to represent pure (B97D3 and PBE-D3BJ) and hybrid (CAM-B3LYP-D3BJ, ωB97XD, and M06-2X), dispersion corrected and noncorrected, and global and range-separated hybrid. For these functionals, the aug-cc-pVTZ calculations presented in Section 3.1 were repeated with two quadruple-ζ basis sets: aug-cc-pVQZ and def2-QZVPP. Table 7 shows the MAE for the total and three-body interaction energies for the 10 global minimum structures. Differences between the aug-cc-pVTZ and aug-cc-pVQZ basis set results are small: less than 0.05 kcal/mol for total interaction energies and less than 0.02 kcal/mol for three-body interactions in most cases. The only slightly larger differences come from using the def2-QZVPP basis set with the B97D3 and PBE-D3BJ functionals. In these cases, accuracy is slightly decreased except for B97D3 total interactions, for which the accuracy increases slightly. Thus, aug-cc-pVTZ can be taken to be a relatively complete basis set which describes the electron density well for total and three-body interactions within the margin of 0.05 kcal/mol for this data set.^2^

**Table 7:** MAE for Total Interaction and Three-Body Interaction Energies for a Benchmark Set of 10 Global Minimum Molecular Complexes Calculated with Five DFT Functionals and Three Basis Sets, Compared to the CCSD(T) Reference Values^2^^a^

	aug-cc-pVTZ		aug-cc-pVQZ		def2QZVPP	
	*E*_int_	*E*_int_^3^	*E*_int_	*E*_int_^3^	*E*_int_	*E*_int_^3^
M062X	0.6	0.344	0.524	0.364	0.557	0.332
CAM-B3LYP-D	0.803	0.108	0.824	0.110	0.826	0.125
B97D3	0.932	0.203	0.913	0.206	0.866	0.23
PBE-D3BJ	0.783	0.245	0.805	0.246	0.903	0.274
ωB97XD	0.585	0.181	0.573	0.196	0.545	0.176

a Values are in kcal/mol.

### 3.3 Local Minimum Structures

The same five functionals used in basis-set convergence testing (CAM-B3LYP-D3BJ, M06-2X, B97D3, ωB97XD, and PBE-D3BJ) were used to calculate the total and three-body interactions for the local-minimum structures from the reference data set.^2^Table 8 shows the interaction energy values, and Table 9 shows the MAE for the 10 complexes. The magnitudes of the errors in all cases are similar to the errors for the global minimum structures (Table 6). The M06-2X, ωB97XD, and B97D3 functionals are slightly more accurate for the local-minimum structures, while the CAM-B3LYP-D3BJ functional is slightly less accurate. The PBE-D3BJ functional is less accurate for the total interactions of the local-minimum structures and more accurate for the three-body interactions. Since the local-minimum structures are less strongly bound than the global minima, the increased accuracy from the M06-2X, ωB97XD, and B97D3 functionals suggests that they can model the longer-range and weaker forces more accurately. This conclusion is also supported by the fact that they are two of the three most accurate functionals for total interactions in this work. Although all five of the functionals in this section were selected due to good accuracy for global minima, the only functional among them that has both a low total interaction error and a low three-body interaction error is ωB97XD.

**Table 8:** Total Interaction and Three-Body Interaction Energies for a Benchmark Set of 10 Local Minimum Molecular Complexes Calculated with Five DFT Functionals and the def2-QZVPP Basis Set, Compared to the CCSD(T) Reference Values^2^^a^

	reference^2^		CAM-B3LYP-D3BJ		M06-2X		B97XD		PBE-D3BJ		ωB97XD	
	*E*_int_	*E*_int_^3^	*E*_int_	*E*_int_^3^	*E*_int_	*E*_int_^3^	*E*_int_	*E*_int_^3^	*E*_int_	*E*_int_^3^	*E*_int_	*E*_int_^3^
CH_3_I–H_2_CO–H_2_O	–7.04	–0.57	–7.77	–0.66	–6.81	–0.72	–6.68	–0.70	–8.08	–0.63	–6.30	–0.76
CF_3_Cl–H_2_CO–H_2_O	–8.69	–0.56	–9.36	–0.62	–8.40	–0.70	–7.08	–0.61	–8.67	–0.52	–7.54	–0.71
CH_3_OH–H_2_O–H_2_O	–14.12	–1.59	–15.49	–1.73	–14.06	–1.78	–12.50	–1.81	–14.82	–1.66	–13.89	–1.85
NH_4_^+^–C_6_H_6_–H_2_O	–32.39	–4.62	–34.21	–4.88	–33.96	–4.53	–33.05	–5.01	–34.59	–5.06	–33.42	–4.59
Cl^–^–NH_3_–C_6_H_6_	–11.80	1.17	–11.26	1.27	–12.17	1.03	–11.69	1.45	–12.53	1.68	–11.12	1.18
C_6_H_5_Br–(CH_3_)_3_N–C_6_H_6_	–7.33	–0.12	–6.24	–0.23	–6.82	–0.73	–7.55	–0.19	–6.83	–0.04	–7.07	–0.37
C_6_H_5_OH–C_6_H_6_–H_2_O	–6.25	–0.01	–5.05	–0.03	–6.48	0.01	–6.65	–0.03	–5.72	–0.02	–6.18	–0.01
C_6_H_5_Cl–C_6_H_6_–NH_4_^+^	–32.96	4.15	–33.47	4.48	–34.30	3.84	–35.09	4.66	–34.85	4.91	–33.72	4.13
HCCH–C_6_H_6_–H_2_O	–7.39	–0.51	–7.40	–0.57	–7.98	–0.90	–7.15	–0.54	–7.73	–0.43	–7.21	–0.69
HCCH–C_6_H_6_–C_6_H_6_	–4.92	0.06	–4.18	0.00	–4.54	–0.36	–5.82	0.04	–4.93	0.16	–5.15	–0.13

a Values are in kcal/mol.

**Table 9:** MAE for Total Interaction and Three-Body Interaction Energies for a Benchmark Set of 10 Local Minimum Molecular Complexes Calculated with Five DFT Functionals and the def2-QZVPP Basis Set, Compared to the CCSD(T) Reference Values^2^^a^

	*E*_int_	*E*_int_^3^
M062X	0.554	0.247
CAM-B3LYP-D	0.868	0.123
B97D3	0.825	0.171
PBE-D3BJ	0.795	0.215
ωB97XD	0.533	0.128

a Values are in kcal/mol.

### 3.4 Three-Body (ATM) Empirical Dispersion Corrections

Three test complexes were chosen to evaluate the effects of the ATM three-body dispersion terms^28,36^ in the calculation of the total and three-body interaction energies (see Table 10). These complexes were chosen to represent dipole–dipole–dipole interactions, dipole–dipole–quadrupole interactions, and quadrupole/quadrupole/quadruple interactions. They also include a relatively large attractive three-body interaction, a large repulsive three-body interaction, and a near-zero three-body interaction, respectively. The PBE functional was chosen as the test functional, as it serves to isolate the dispersion contribution. The pure PBE and hybrid HSE functionals have poor performance for total interaction energies, but the addition of the D3BJ dispersion corrections to the PBE functional improves the total interaction energy. The addition of HF exchange in the HSE functional, on the other hand, improves the three-body interaction energy by about 50%, but has a much smaller effect on the total interaction energy. Thus, by studying the ATM addition to D3BJ in this case, the three-body dispersion corrections can be evaluated in the absence of nonadditive exchange. The def2-QZVPP basis set was chosen, as it is smaller but offers similar accuracy to aug-cc-pVQZ.

**Table 10:** Total Interaction and Three-Body Interaction Energies for Three Sample Complexes (Global Minima) Calculated with PBE-D3BJATM and the def2-QZVPP Basis Set, Compared to the CCSD(T) Reference Values^2^^a^

	reference		PBE-D3BJ		PBE-D3BJATM		3 body dispersion	
	*E*_int_	*E*_int_^3^	*E*_int_	*E*_int_^3^	*E*_int_	*E*_int_^3^	*E*_int_	*E*_int_^3^
CH_3_OH–H_2_O–H_2_O	–16.99	–2.42	–18.01	–2.55	–18.00	–2.54	0.01	0.01
NH_4_^+^–C_6_H_6_–H_2_O	–36.23	3.39	–38.25	3.97	–38.18	3.98	0.07	0.01
HCCH–C_6_H_6_–C_6_H_6_	–6.95	–0.20	–6.64	–0.10	–6.50	–0.05	0.14	0.05

a The final column is the difference between the two-body corrected dispersion energies (PBE-D3BJ) and the three-body corrected energies (PBE-D3BJATM). Values are in kcal/mol.

Table 10 shows the ATM-corrected PBE-D3BJ values as well as the difference between PBE-D3BJATM and PBE-D3BJ. The last column gives the isolated ATM three-body dispersion contributions. The size of the three-body dispersion contribution to the total interaction energy does not correspond to the size of the total interaction; for example, the largest three-body dispersion contribution comes on the smallest total interaction energy. The three-body dispersion contributions are at most ∼2% of the total but are as small as 0.2% of the total and, in all cases, at most one order or magnitude smaller than the *average error* for that method. The sizes of the three-body dispersion contributions to the three-body interactions likewise do not correlate to the sizes of the three-body interactions, and they are smaller in magnitude than the three-body dispersion corrections to the total energy. They do make up in one case 25% of the reference value for three-body interactions, but for the rest of the cases they are less than 0.5% of the reference value. It is notable that the case for which the three-body dispersion corrections are the largest are for the quadrupole/quadrupole/quadrupole, or π–π–π interactions. In this case, dispersion is a much larger contributor to the total since there are no dipole-based interactions.

## 4 Conclusions

This work has examined the total and three-body interaction energies for a range of different types of DFT functionals with differences in the amount and use of HF exchange, in the use of empirical dispersion corrections, and in physical origins/calibration. DFT results were compared to CCSD(T)/CBS results.^2^ It was found that inclusion of HF exchange, via either a global or range-separated hybrid approach, increases accuracy for both total and three-body interactions. For total interactions, this is primarily due to the nonlocal nature of HF exchange, but for three-body interactions, this is primarily due to the inclusion of nonadditive exchange in HF that is not present in pure-DFT functionals. In lieu of HF exchange, empirical dispersion corrections can also contribute to the accuracy of the total interaction energy of a functional, although in some cases, the addition of empirical dispersion to HF exchange does not improve accuracy further. For three body-only interactions, the addition of empirical dispersion contributes only slightly unless the dispersion correction includes three-body interactions explicitly (such as with the D3ATM method). The aug-cc-pVTZ basis set used with the functionals studied here provides good accuracy, and testing with aug-cc-pVQZ and def2-QZVPP shows that aug-cc-pVTZ is fairly well converged, i.e. little to no change is seen with quadruple-ζ basis sets. Similar results are obtained when studying global minimum and local minimum complex structures. A model chemistry of aug-cc-pVTZ with CAM-B3LYP-D3BJ, B97D3, or ωB97XD is recommended for calculating three-body interactions.

## Supporting Information Available

Raw data for counterpoise-corrected interaction energies for all DFT methods and basis sets studied here (XLSX)
